# The Effect of Immersive Virtual Reality on Anterograde Amnesia and Subjective Pain During Procedures: A Within-Subject Randomized Controlled Study

**DOI:** 10.1213/ANE.0000000000007858

**Published:** 2026-05-15

**Authors:** Hunter G. Hoffman, Herta Flor, Brett R. Stacey, Iaroslav Tsymbaliuk, Beatrice Addai, Keira P. Mason

**Affiliations:** From the 1 Virtual Reality Research Center, Human Photonics Lab, Department of Mechanical Engineering; 2 Center for Human Neuroscience, Department of Psychology, University of Washington, Seattle, Washington; 3 Department of Neuropsychology and Psychological Resilience Research, Research Group Learning, Brain Plasticity and Mental Disorder, Central Institute of Mental Health, Medical Faculty Mannheim, Heidelberg University, Heidelberg, Germany; 4 Department of Anesthesiology and Pain Medicine, University of Washington, Seattle, Washington; 5 State Institution “Romodonov Neurosurgery Institute of the National Academy of Medical Sciences of Ukraine”, Kyiv, Ukraine; 6 Peace and Love Hospitals Kumasi and Accra, Kumasi, Ghana, Africa; 7 Department of Anesthesiology, Critical Care and Pain Medicine, Harvard Medical School, Boston Children’s Hospital, Boston, Massachusetts.

## Abstract

**BACKGROUND:**

Immersive virtual reality (VR) distraction reduces procedural pain. The current study explores whether immersive VR also reduces how much people can remember about a painful experience: anterograde amnesia for pain.

**METHODS:**

A within-subject, crossover design was used. Sixteen healthy adult volunteers participated. Each participant received 5 thermal stimuli (some hot, some cold) during no-VR and 5 stimuli during immersive VR distraction (treatment order randomized). They were instructed to memorize the order of hot and cold stimuli for a later memory test. After a brief delay after each stimulus set, participants completed a memory recall test for the order of stimuli (the primary measure) and also provided ratings of pain, distraction, anxiety, and nausea using standardized graphic rating scales (GRS; 0–10).

**RESULTS:**

Within-subject Wilcoxon signed-rank tests revealed that immersive VR significantly reduced how accurately participants could recall the hot/cold order of thermal stimuli, mean accuracy: 96% correct (standard deviation [SD] = 8.06) in no-VR, versus 59% (SD = 25.79) in VR, *Z* = 3.09, *P* = .002, *r* = 0.77. On a rating scale from 0 to 10, immersive VR was significantly more distracting (mean = 7.60, very distracting, SD =1.72) compared to the control condition (mean = 2.07, mildly distracting, SD = 2.16, *Z* = 3.42, *P* < .001, *r* = 0.86), and on GRS mean pain perception ratings, participants reported significantly lower pain intensity during VR, mean = 4.03 (SD = 1.61) during VR, versus no-VR = 6.30 (SD = 1.81), *Z* = 3.47, *P* < .001, *r* = 0.87.

**CONCLUSIONS:**

Results of this study provide preliminary evidence that immersive VR reduces memory for a painful experience. Conscious/episodic memory formation and storage of memories about specific experiences requires attentional resources. VR distraction pain intervention significantly disrupts memory for painful stimuli, leading to what we term “VR amnesia.” Our study provides preliminary laboratory evidence that immersive VR during pain induces anterograde amnesia for pain, disrupting the formation of memory for painful events. Further studies exploring the mechanism of how VR reduces memory for painful events are needed. These results suggest the utility of future studies in clinical pain contexts. If VR can reduce the formation of adverse memories associated with painful clinical procedures, VR may serve as an effective nonpharmacological adjunct to reduce postoperative distress and medications and may reduce risk of developing chronic pain.

## Graphical abstract

**Figure:**
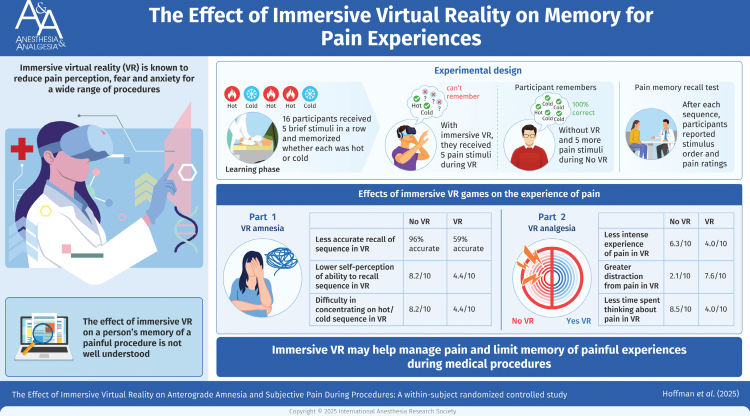

**KEY POINTS**

- **Question:** Does immersive virtual reality reduce how much a person can later remember about a painful event?
- **Findings:** Immersive virtual reality significantly reduced memory for pain.
- **Meaning:** Immersive virtual reality appears to interfere with memory encoding/storage of nociceptive information, and may have clinical value as a nonpharmacologic amnesic.

The National Institute of Health (NIH) has launched several initiatives to encourage the development of nonpharmacological techniques to minimize pain.^1^ Virtual reality (VR) is proving effective for reducing pain perception, fear, and anxiety for a wide range of painful procedures,^2–16^ but little is known about the effect of immersive VR on memory for pain. The current study measured whether immersive VR reduces how much a person can remember about a painful experience. Conscious/episodic memory formation about specific experiences is known to require attentional resources.^17–19^ Encoding is the process of transferring raw sensory information into a form that the brain can process and store. If information is not encoded effectively, it is less likely to be remembered or consolidated into long-term memory. We predict that VR divides consciousness, leaving the person partly conscious in the real world and largely conscious in the virtual world (Figure 1). VR limits the amount of attention participants have available to encode and store new non-VR information (eg, from pain receptors) in their brain, reducing people’s memories for pain they experience in the real world while they are in VR. If VR can reduce the formation of adverse memories of pain experienced during medical procedures, “VR amnesia” could have significant clinical implications. This study systematically explores whether VR induces anterograde amnesia for pain experienced while in VR. The study is designed to add to our understanding of how immersive technology can impact pain perception and memory.

**Figure 1.**
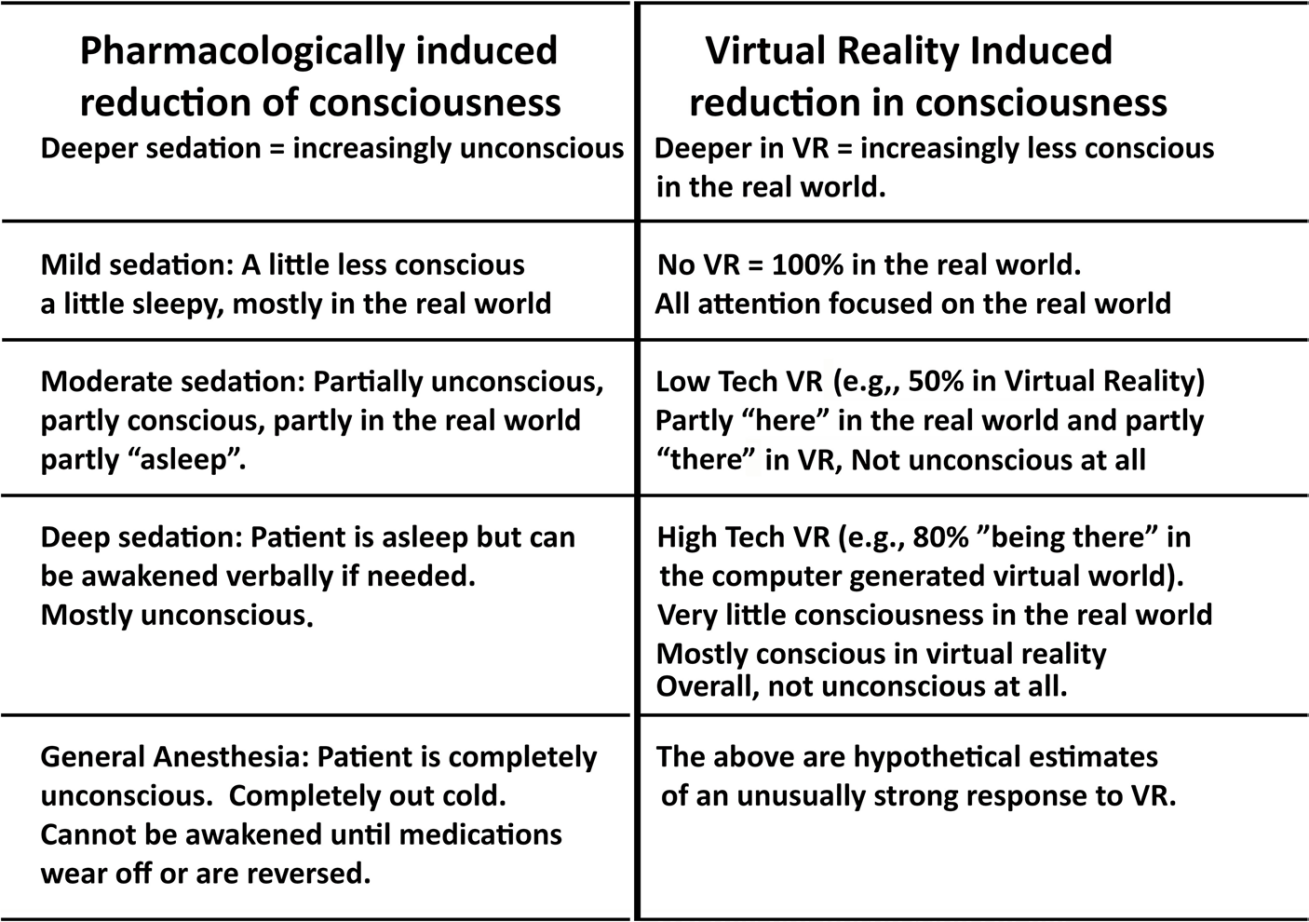
Traditional sedation results in pharmacological reductions in consciousness. Virtual reality divides consciousness, leaving the person partly conscious in the real world and largely conscious in the virtual world. VR indicates Virtual Reality.

**Table 1.** Inclusion and Exclusion Criteria for Study Participation

Inclusion criteria
• Currently enrolled in a course at the Psychology Department, University of Washington, participating in the UW Psychology subject pool
• Able to read, write, and comprehend English
• Able to complete study measures
• Willing to follow our UW-approved instructions
• ≥18 yr of age
Exclusion criteria
• People who have already previously participated in this same study (eg, last quarter) are not eligible to participate again.
• Not enrolled in a course at the Psychology Department, University of Washington, not participating in the UW Psychology subject pool
• Not be able to read, write, and comprehend English
• <18 yr of age
• Not capable of completing measures
• Not capable of indicating pain intensity
• Not capable of filling out study measures
• Extreme susceptibility to motion sickness
• Seizure history
• Unusual sensitivity or lack of sensitivity to pain
• Sensitive skin
• Sensitive feet
• Migraines
• Diabetes

From Hoffman 2021,^21^ licensed under CC BY-SA 4.0 (creativecommons.org/licenses/by-sa/4.0/).

Abbreviation: UW, University of Washington.

**Figure 2.**
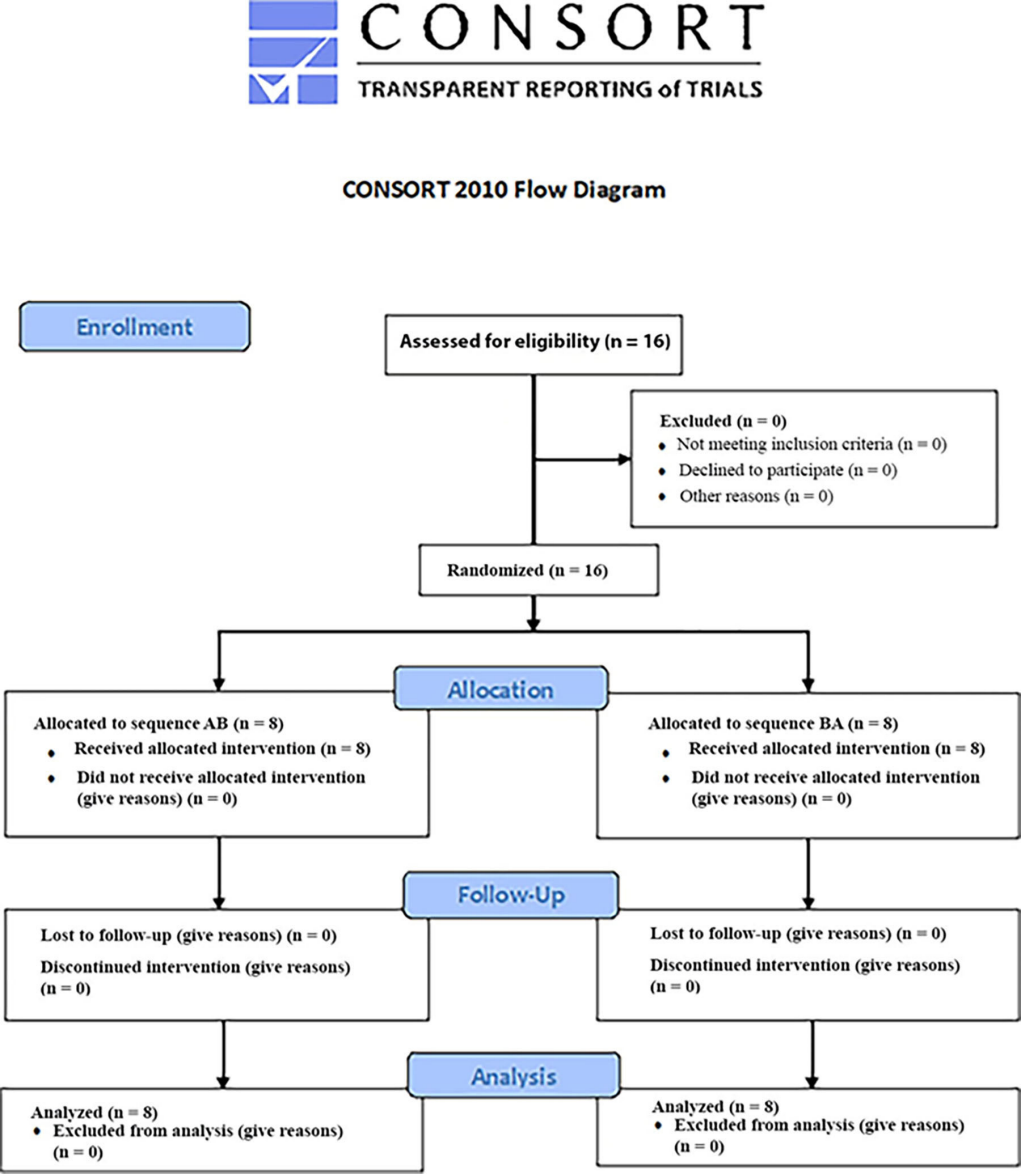
Consolidated Standards of Reporting Trials flow diagram depicting participant progression through eligibility assessment, randomization, intervention sessions, and analysis, including reasons for withdrawal in the VR-induced amnesia for pain trial. VR indicates Virtual Reality.

**Figure 3.**
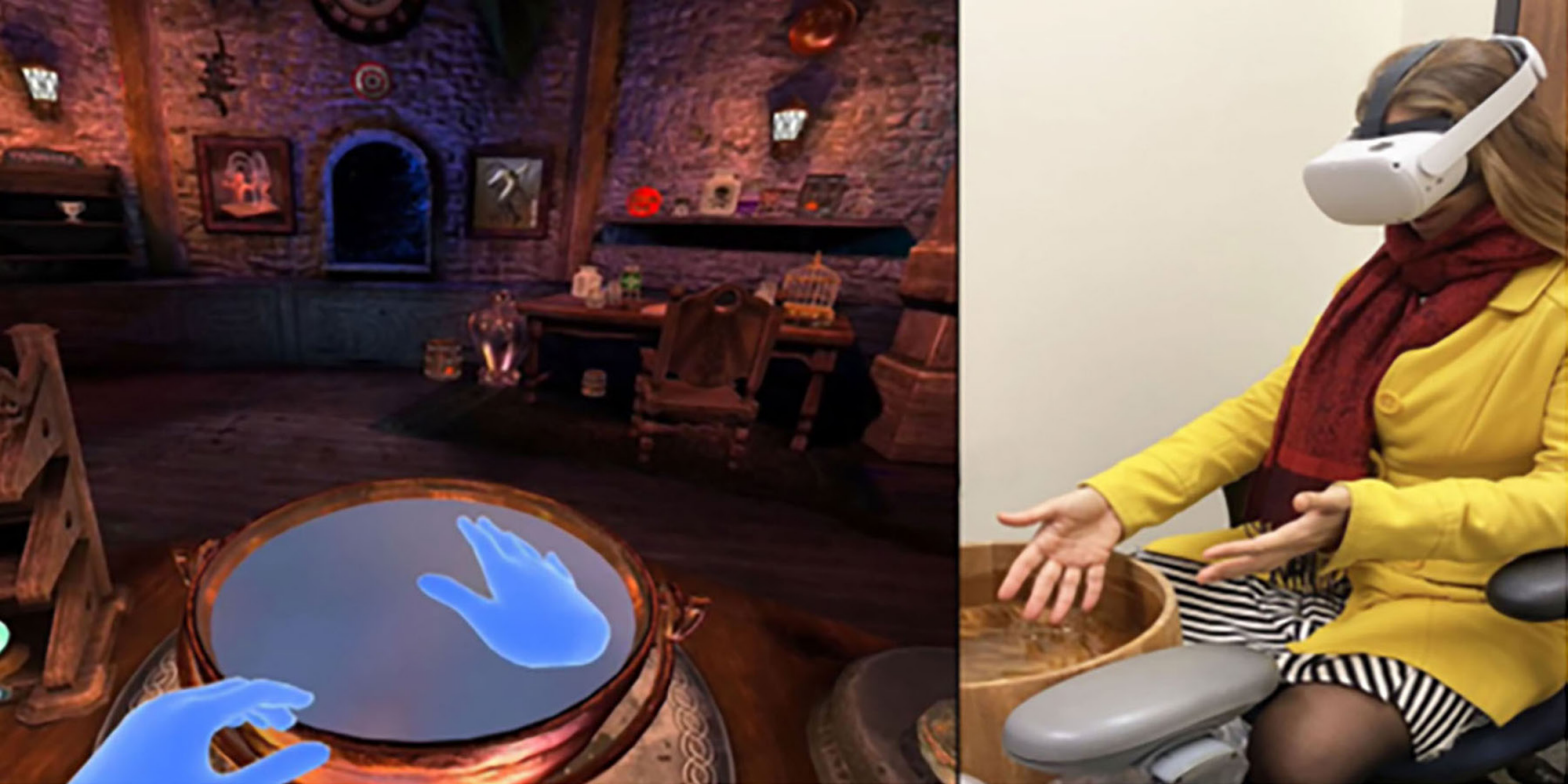
Mixed reality. VR  +  real water. Using camera-based hand tracking, participants interacted with virtual water via their cyberhands and cyberfingers. A bowl of real water was used to give the virtual water physical qualities; warm, wet, and resistant to stirring (image on the right, photo and copyright Hunter Hoffman, UW, www.vrpain.com). The image on the left is from www.aldin.io/waltzofthewizard; Reykjavik, Iceland, used with permission. Left, A screenshot from Waltz of the Wizards VR world (image by www.aldin.io, used with written permission). Right, A photo of a participant in virtual reality using our VR pain intervention known as the “magic bowl,” a form of mixed reality, which uses real water to make virtual water seen in immersive VR world more distracting (photo and copyright Hunter Hoffman, www.vrpain.com). Participants used their cyber hands to put virtual objects into the magic bowl, to make magic spells. VR indicates Virtual Reality.

## METHODS

### Study Design and Population

A within-subject, crossover design was used. Sixteen healthy adult volunteers from the University of Washington (age 18–42 years old, mean age = 23.25, standard deviation [SD] = 6.03, 62.5% female, 63% Asian, 37% White) participated. Ethics committee (institutional review board [IRB]) approval was obtained from the IRB of the University of Washington Committee B, and written informed consent was received from all participants. The study protocol adhered to the Declaration of Helsinki. The inclusion and exclusion criterion were delineated (Table 1). All data were collected at the University of Washington, Seattle, in a laboratory room designated for VR analgesia research studies, during a single 50-minute visit (ClinicalTrials.gov ID NCT06871423, completed January 3, 2025). None of the participants were harmed and none experienced unintended effects. See Consolidated Standards of Reporting Trials diagram in Figure 2.

### Equipment

The immersive VR system for this study was an untethered self-contained battery powered stand-alone wireless MetaQuest2 Head Mounted Display VR system (firmware build 72). Optical video camera–based hand tracking (Meta, www.meta.com) with optional passive hardware controllers were also available for the researcher during setup. The immersive VR software used was a commercially available VR game named Waltz of the Wizards (www.aldin.io/). In the fully immersive VR treatment condition, each participant could see a medieval room in a castle in VR, with a large virtual wooden bowl full of computer-generated animated virtual water within reach of their virtual hand. Cameras embedded in the VR helmets were used to track the movements of the participants’ dominant hand. This allowed participants to use their real hand movements of their dominant hand to make their cyberhand grab virtual objects and put the virtual objects into the magic bowl, to make magic spells (Figure 3). Our previous studies on VR, pain, and attention have measured/quantified that this VR pain intervention is attention demanding.^20^

### Quantitative Sensory Testing

To simulate a painful medical procedure (eg, venipuncture) in a way that allowed us to measure pain, we developed an original Quantitative Sensory Testing (Medoc Ltd, 2024) paradigm for the current study. Brief hot and cold stimuli (interleaved) were delivered via a Q-Sense CPM thermal stimulator from Medoc LTD, Israel, using the Medoc Advanced Medical Systems Q-sense https://www.medoc-web.com/q-sense method of ascending levels. The thermode was affixed to the ventral wrist of the participant’s nondominant hand.

### Procedure

Each participant used the “method of limits” to select the “painful but tolerable” temperature of the stimuli they would receive in this study. The temperature slowly rises for warm stimuli or slowly falls for cold stimuli, and participants push a “stop” button when the temperature becomes “painful but tolerable” at a temperature they are willing to receive several more times later in the study. When the “stop” button is pushed by the participant, the temperature immediately begins returning to baseline temperature. Participants were told we were ideally looking for a peak “worst pain” level around 5 or 6 on a scale from 0 to 10, but they could push the button to select any temperature they wanted. The mean hot “painful but tolerable” stimulus temperature selected by participants in the current study was 39.79 °C (range = 33 °C–46.10 °C). The mean painful but tolerable cold stimulus temperature selected by participants was 22.57 °C (range = 16 °C–29 °C, where 16 °C was the coldest possible for our equipment). Once the participant selected the temperature, the researcher controlled all subsequent stimulus presentations at the temperatures the participant preapproved.

**Figure 4.**
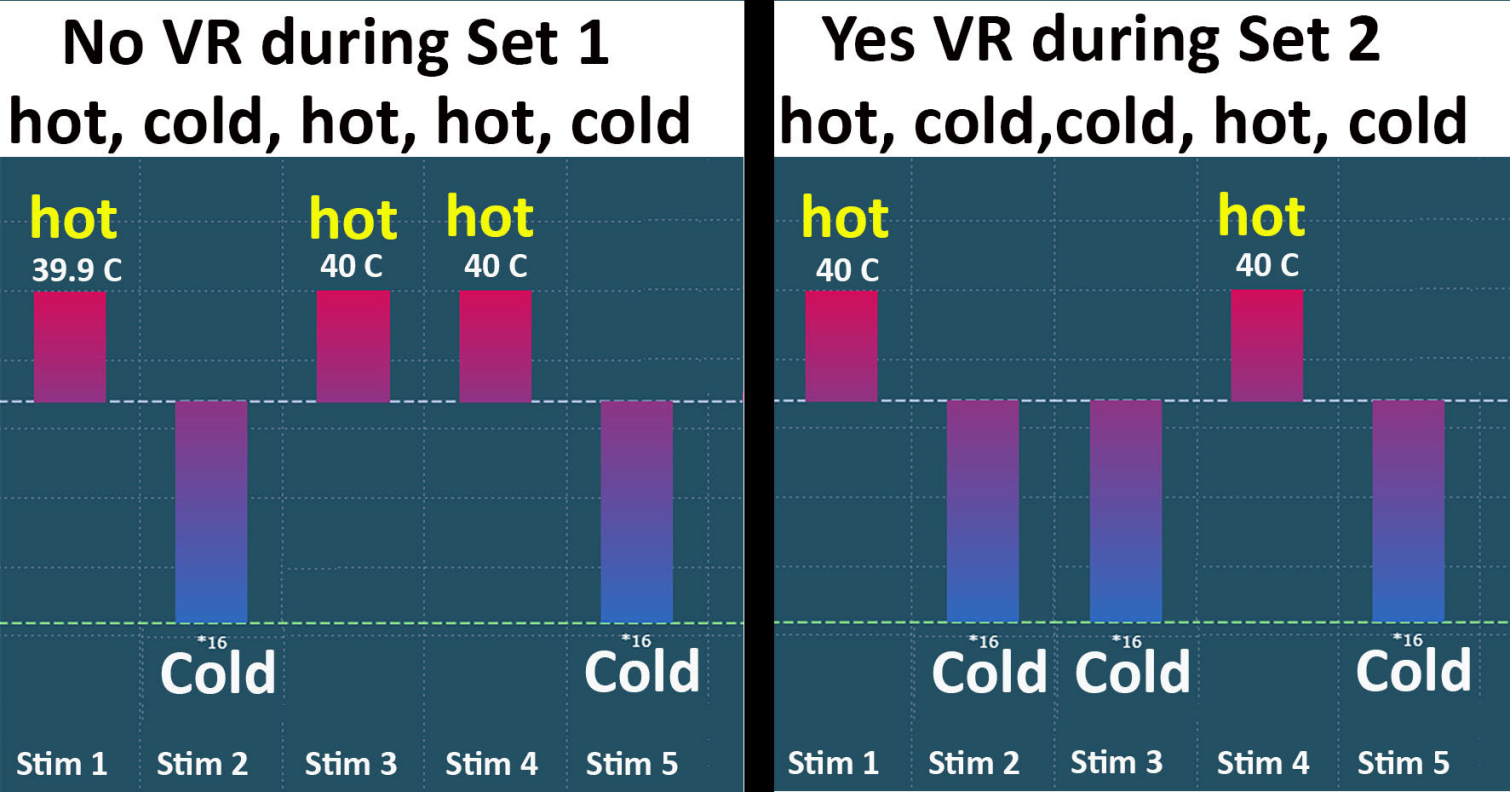
Participants were instructed to deliberately memorize the exact pattern of a total of 5 painful stimuli per set (some hot, some cold), eg, “was stimulus 1 hot or cold?, was stimulus 2 hot or cold?, etc. Participants memorized the order of hot and cold stimuli during no VR in one set, and during immersive virtual reality with tactile feedback during the other set (treatment order randomized). Left, Before set 1, this participant selected 40°C as her preferred hot stimulus and 16°C as her preferred cold stimulus. She then received 5 stimuli in the sequence shown and completed an immediate pain-memory recall test (correct responses: hot, cold, hot, hot, cold). Right, In set 2, dive stimuli in a different order. We hypothesized poorer memory for the stimulus sequence experienced during immersive VR (order of VR versus no-VR sets randomized across participants). VR indicates Virtual Reality.

The primary outcome measure was the participants’ accuracy of deliberately memorizing the exact pattern of a total of 5 pain stimuli per set (some hot, some cold). As mentioned briefly earlier, using the Quantitative Sensory Testing “method of limits,” each participant selected a warm and a cold temperature they found “painful but tolerable” that they were willing to receive several more times, later in the study. They then received a sequence of 5 stimuli to their wrist (controlled by the researcher), with a brief interstimulus interval between each stimulus. All participants received hot (H) and cold (C) stimuli in the following order (HCHHC) for set 1, and all received a slightly different order (HCCHC) for set 2 (Figure 4). Before each sequence, participants were told that they would feel 5 brief thermal stimuli, some hot, some cold, 5 total. Participants were instructed that their task was to memorize the sequence of painful stimuli they were about to experience, and their memory for the painful stimuli would be tested later.

### Instructions to Each Participant

“You will now receive five thermal pain stimuli, some hot, and some cold stimuli (with brief delays between each stimulus). Afterwards, you will be given the following memory test. You will be asked to remember if stimulus 1 was hot or cold?, was stimulus 2 hot or cold?, was stimulus 3 hot or cold?, was stimulus 4 hot or cold?, was stimulus 5 hot or cold?, and you will be asked to rate how painful you found the overall pain stimulus set.

### Pain Memory Test

To introduce a brief delay between study and recall test, participants performed a numeric distractor task (the odd number task)^20,21^ for 15 seconds immediately after experiencing the brief hot/cold sequence of 5 painful stimuli (eg, HCHHC for stimulus set 1). After the odd number task, participants were asked to recall the exact pattern of the most recent sequence of 5 stimuli (hot and cold brief thermal stimuli), recalled in exact order, via our 5 question “pain memory recall test” briefly described earlier. If they were completely guessing on any of the answers (eg, if they are drawing a blank about whether stimulus 4 was hot or cold), they could leave a question mark for that answer. Unbeknownst to the participants, question mark responses from participants were later scored as incorrect. After the pain recall memory test, they were then asked the subjective ratings shown in Supplemental Digital Content 1, https://links.lww.com/AA/F599.

After completing the above measures for the first stimulus set, the researcher then briefly reread the instructions to the participant, and the participant experienced a second, slightly different brief hot/cold pain stimulus sequence 2 (HCCHC) while simultaneously in VR interacting with objects in a commercially available VR game named Waltz of the Wizards (www.aldin.io/). After VR, they then performed a brief (15 seconds) numeric odd number task, monitoring a string of numbers and saying “now” any time they heard 3 odd numbers in a row,^20^ followed by the primary outcome measure, the pain recall memory test described earlier. Participants then filled out graphic rating scale (GRS) pain and presence ratings, and a Global Impression of Change measure.^22^ Participants were asked to report their global impression of change on a 7 point scale from −3 (very much worse) to + 3 (very much better) (see questionnaire in Supplemental Digital Content 1, https://links.lww.com/AA/F599).

Treatment order (no-VR first versus VR first) was randomized using computer-generated random number sequences from random.org, with block size of 2. To reduce selection bias, we generated an unpredictable pattern of 1’s and 2’s and concealed this sequence from the investigator enrolling participants. Sequentially numbered opaque envelopes were used to conceal the treatment order until the participant had arrived and was consented to participate. The first author enrolled the patients and assigned them to the randomized treatment order. All participants were included in the analysis in the treatment order to which they were originally assigned (intention-to-treat analysis). Outcomes were separately analyzed by an independent statistician who was kept blind to the treatment conditions.

### Measures

The primary outcome measure was the participant’s ability to deliberately memorize and remember the exact sequence of 5 hot and cold painful stimuli, in the exact order, as instructed before each pain stimulus set. In addition, participant’s subjective ratings of the accuracy of their memory for the painful stimuli was then measured on a GRS, and they answered a single item Global Impression of Change measure.^22^

### Secondary Outcome Measures

Each participant also later rated their subjective experiences and their overall pain during VR versus during no-VR, using GRS validated by the measures’ strong associations with other measures of pain intensity, as well as through the measure’s ability to detect treatment effects.^23,24^ GRS ratings were used to measure worst pain, pain unpleasantness, and time spent thinking about pain that correspond to three separable components of the pain experience; sensory pain, affective pain, and cognitive pain, respectively (Supplemental Digital Content 1, https://links.lww.com/AA/F599). The question regarding “to what extent did you feel like you ‘went into’ the virtual world,” was adapted from Slater et al.^25,26^ Similar presence measures have been used to establish reliable metrics for the effect of treatment.^21^ Participants also filled out the following 3 GRS measures: “How accurate is your memory for the painful stimuli during the most recent pain stimulus set (5 painful stimuli in a row),” “How distracted were you during the most recent pain stimulus set (5 painful stimuli in a row), while trying to memorize the pattern of pain stimuli,” and “How hard was it to concentrate on memorizing the pattern of thermal pain stimuli during the most recent pain stimulus set (5 painful stimuli)” (Supplemental Digital Content 1, https://links.lww.com/AA/F599).

### Power Analysis

A power analysis to determine the number of participants needed to test our primary hypothesis was computed using the statistical program GPower 3.10. The following assumptions were used in the power analyses, all determined from the divided attention task by Hoffman et al 2023,^20^ an effect size (*d*) of 0.83, power of 0.80, and an *α* of .05. Under these conditions, we would detect a significant ability of immersive VR to affect the recall of painful stimuli experienced during their immersion.

## RESULTS

Participants’ actual accuracy of memory for the exact sequence of hot and cold pain stimuli received during no-VR versus during VR was analyzed using a within-subject nonparametric paired comparison Wilcoxon signed-rank test. In each set, there are 5 pain stimuli and 5 possible correct answers, 5/5 = 100% accurate, if the participant incorrectly said “hot” to one of the cold stimuli (or incorrectly said “cold” to one of the hot stimuli), they would get 4/5 = 80% accurate, etc. As predicted, participants were significantly less accurate in remembering the correct hot and cold order of the 5 pain stimuli they experienced in VR (59% accurate, SD = 25.79) versus the correct hot and cold order of the 5 pain stimuli participants received during no-VR (96% accurate, SD = 8.06), *Z* = 3.09, *P* = .002, *r* = 0.77 a large effect size. In addition, participants were subjectively well aware that their memory for pain during VR was less accurate. On a subjective rating scale from 0 to 10, participants rated how accurately they had recalled their pain in each treatment condition. On the GRS, participants reported/estimated they had been “pretty accurate” during no-VR (8.2 on a 0 to 10 scale, SD = 1.86) and participants estimated that they had been only “mildly accurate” during immersive VR (4.40 of 10, SD = 1.85), *Z* = 3.42, *P* < .001, *r* = .86 a large effect size, using a Wilcoxon signed-rank test (paired samples). The global impression of change measure showed a similar pattern. On average, participants reported that the accuracy of their memory for the painful stimuli was much less accurate during VR than during no-VR.

On the GRS from 0 to 10, participants reported that while trying to memorize the hot and cold thermal pain stimuli order, during no-VR they were only “a little distracted” (mean = 2.07, SD = 2.16), and, on average, during the fully immersive magic bowl VR condition, they were “pretty distracted” (mean = 7.60, SD = 1.72), using Wilcoxon signed-rank test, *Z* = 3.42, *P* < .001, *r* = 0.86.

Similarly, on (0–10) subjective ratings, participants reported that it was only “a little” difficult/hard to concentrate on the thermal pain stimuli during no-VR (mean = 2.33, SD = 2.19) and it was “very difficult/hard to concentrate on memorizing the order of hot and cold thermal pain stimuli they received during the fully immersive Magic Bowl “Waltz of the Wizards” VR (mean = 7.70, SD = 1.49), *Z* = 3.41, *P* < .001, *r* = 0.85, a large effect size.

### Exploratory Results on Whether Higher Presence Leads to Greater VR Amnesia

Although the sample size was not large enough to conduct formal statistics, participants showed the predicted pattern (Supplemental Digital Content 1, Figure A, https://links.lww.com/AA/F599). Participants who reported a strong illusion of “being there” in the computer-generated world as if it was a place they visited showed large VR-induced amnesia: 50% reductions in pain memory accuracy, and they reported that it was very hard/difficult to concentrate on their pain during VR. In contrast, the subset of participants who reported only “mild to moderate” presence during VR showed only a 23% reduction in pain memory accuracy during VR, and they reported that it was only moderately difficult to concentrate during VR.

In addition to measuring reductions in memory for pain (VR amnesia), VR analgesia (reduction in ratings of subjective pain intensity) was also measured by comparing pain during immersive VR in the magic bowl versus pain ratings during no-VR, using Wilcoxon signed-rank tests using SPSS 29.02. As shown in Table 2, using GRS pain ratings, participants reported a significant reduction in several measures of pain during the brief thermal stimuli set (of 5 brief stimuli) on traditional measures of VR analgesia (no-VR versus immersive VR).

**Table 2.** Pain Ratings During a Set of 5 Brief Thermal Stimuli in No-VR Versus Immersive VR Conditions

	No-VR Versus Immersive VR	Wilcoxon *Z* scores	*P* values	Wilcoxon signed-rank Effect size
Time spent thinking about pain	8.50 (2.01) vs 4.00 (1.78)	3.41	<.001	*r* = 0.85
Pain unpleasantness	5.40 (2.44) vs 3.50 (1.90)	2.83	.005	*r* = 0.71
Worst pain intensity	6.30 (1.81) vs 4.03 (1.61)	3.47	<.001	*r* = 0.87
Fun	2.20 (2.73) vs 6.60 (2.77)	2.59	.01	*r* = 0.65
Anxiety	3.60 (2.20) vs 3.30 (2.43)	<1.00	>.05 NS	*r* = 0.11

Abbreviations: NS, non-significant; VR, virtual reality.

Specifically, on these secondary pain measures, as shown in Table 2, during immersive VR, participants reported significant reductions in time spent thinking about pain during the stimulus, pain unpleasantness, worst pain intensity, and they had significantly more fun during immersive VR, compared to no-VR. On a 0 to 10 scale measuring “presence,” where 0 = I did not go inside at all, 5 is a moderate sense of going inside, and 10 = I went completely inside the computer-generated world, participants reported a moderate illusion of going inside the computer-generated world as if it was a place they visited (mean = 5.59, SD = 1.86). The simulator sickness ratings mean was 0.14, SD = 0.53, range from 0 to 2 on a 10-point scale. On a 0 to 10 scale, 1 participant rated their simulator sickness as 2 of 10 (mild nausea), and all other participants rated their simulator sickness from VR as 0.

## DISCUSSION

Our study provides preliminary evidence that in addition to reducing the pain intensity felt by the participant (VR analgesia), our immersive VR distraction pain intervention significantly disrupts memory for painful stimuli, leading to what we term “VR amnesia.” In the current study, participants demonstrated markedly reduced recall accuracy for the pain they experienced in the real world while in immersive VR. Subjective ratings further support our finding that participants found VR highly distracting, and participants were aware that VR was impairing their ability to later remember the painful events.

Importantly, as further support for our current claims, our findings extend beyond the domain of pain. A related study using a traditional word recall paradigm demonstrated a similar effect: In further support of our distraction hypothesis, preliminary data from a newly completed randomized controlled study showed that immersive VR significantly impaired memory for verbally presented word lists. In a within-subject design, word recall accuracy dropped by 51% in immersive VR compared to a no-VR control, and word recall memory accuracy dropped by 20% relative to a plausible control see-through VR condition. Participants also rated immersive VR as significantly more distracting. These results conceptually replicate our current findings, and the word study^27^ shows that VR-induced amnesia generalizes to non–pain-related episodic memory. Together, these findings suggest that immersive VR disrupts encoding of real-world stimuli across multiple modalities, supporting a broader conceptual model of VR-induced memory disruption.

The underlying mechanisms of how VR reduces memory recall accuracy are unknown, and several mechanisms may be involved. The current results showing VR amnesia for pain are consistent with the involvement of an attentional mechanism, that is, distraction. Encoding and storage of episodic/conscious memories for specific experiences requires a significant allocation of conscious attention.^17,18^ By captivating users’ attention with an alternate, interactive computer-generated environment, immersive VR has been shown to divert these cognitive resources.^7,21^ Consequently, according to our distraction hypothesis, while in VR, people have less attention available to process and store external (non-VR) information, whether painful or verbal, resulting in partial amnesia. Our conceptual framework (Figure 1) posits that while immersed in VR, the combined consciousness allocated to the virtual + the real world still sums to 100%, but VR divides consciousness, drawing attention into VR, limiting the amount of attention available to encode and store new information simultaneously coming into the brain from the real world.

The current results are also consistent with a related “working memory” explanation. Immersive VR may impose a high cognitive load, consuming working memory capacity and leaving fewer resources available for encoding concurrent stimuli such as painful thermal inputs. For example, in one previous laboratory VR study, participants made significantly more errors on an attention demanding “divided attention” task while interacting with the virtual world, compared to passively viewing the virtual world.^21,28^ According to a working memory explanation, the more the person’s attention is drawn into VR, the higher the cognitive load, the less attention they have available for encoding new external information, such as neural signals on their way to the brain from pain receptors in the skin.

Our attentional mechanism “distraction hypothesis” is also compatible with Melzack and Wall’s Gate Control Theory of Pain,^29^ which proposes that the neural transmission of nociceptive signals from nociceptive receptors to the brain is modulated by cognitive and emotional inputs, including attention and positive emotion (fun). Pain memory involves the encoding and storage of pain-related information processed in the brain. If the gate reduces the amount of nociception information allowed into the brain, this could in theory reduce how much pain is remembered in the current study. But the gate control theory is specific to pain, and does not explain how VR reduces memory for word lists in our related study.^27^

The reduced pain recall accuracy (during VR) observed in the current study could also in theory be due in part to memory impairment via a retroactive interference mechanism. Learning new information can disrupt the ability to remember previously learned material, impairing memory retrieval accuracy.^30^

The Contextual Binding Theory of episodic memory^31^ suggests that episodic memory relies on the ability to recall the specific context in which an event occurred, including spatial, temporal, and other details. This theory posits that the hippocampus supports memory by binding together the various contextual elements of an experience. In the context of our study, this theory offers a potential mechanism for how VR may reduce memory for pain. By immersing individuals in a rich, novel virtual environment, VR may disrupt or redirect contextual binding processes away from the nociceptive experience, thereby weakening the episodic encoding of pain memories.

Despite these promising findings, and our recent conceptual replication,^27^ our study has limitations. The sample size is small. The within-subject design, although statistically powerful, does not fully blind participants or outcome assessors to treatment conditions. In research settings, demand characteristics can in some cases bias participants responses. Future studies (eg, with blinding) are needed that use between-group or mixed factorial designs with larger sample sizes to further validate these results and explore the durability of VR-induced amnesia over longer periods and in clinical populations.

It is unknown whether the current results showing VR amnesia (reduced memory) for pain will generalize to clinical settings or for clinically relevant durations. The stimuli used in the current study are deliberately moderate and “painful but tolerable.” With the exception of venipuncture (one of the most common medical procedures), the stimuli used in the current study are too mild and brief to generalize to most surgical or procedural pain settings. Similarly, the current study used healthy volunteers rather than patients. Patients may be more vulnerable to pain or fear of pain.

Regarding generalizability of the current results showing our VR pain intervention reduces memory recall accuracy, encouragingly, recent findings from our related study show that immersive VR also reduces memory for words (VR amnesia for words, no pain involved) and recent laboratory evidence from other related studies quantify that VR is very attention grabbing^20,21^ further supporting the logic of the present findings that VR interferes with memory formation/recall via an attentional mechanism. Although research is needed to determine whether VR amnesia generalizes to clinical settings, encouragingly, in our previous research, the patterns of results on perceived pain ratings found in analog VR analgesia laboratory studies using quantitative sensory testing stimuli have shown the same patterns in clinical studies with severely burned children during 20-minute burn wound care sessions.^3^ Our related previous clinical VR analgesia studies have shown that VR continues to be effective at reducing clinical pain intensity of severely burned children during more prolonged (eg, 20 minute) VR burn wound care sessions, over multiple burn wound care session.^32^

Mason and Hoffman^33^ recently compared and contrasted VR sedation versus traditional pharmacologic sedation, regarding pediatric sedation outside of the operating room. The authors noted that in the scientific literature, pharmacologic sedation and VR sedation both reduce pain and both reduce anxiety. Pharmacologic sedation can also reduce memory for pain. For example, some sedation medications such as benzodiazepines are known amnesic drugs that can be intentionally used to medically reduce patients’ memory for painful medical procedures. Whether VR can also reduce memory for pain is a gap in the literature addressed in the current study.

Similarly, a recent systematic review by Levit et al^34^ (2024) examined 35 randomized controlled trials evaluating the use of virtual and augmented reality (VR/AR) for procedural and postoperative pain relief. This review found that VR has potential for reduced anxiety and sedation requirements in surgical contexts. However, it did not explore how immersive VR might affect memory for pain. The current study addresses this important gap in the literature by introducing and empirically testing the new concept of “VR amnesia.” In addition to reducing pain intensity while in VR, immersive VR significantly disrupted participants’ ability to recall pain afterward. This novel memory-impairing effect may represent an additional mechanism by which VR improves patient outcomes, not only by reducing perceived pain but also by weakening the consolidation of aversive pain memories.

In summary, the current study introduces the notion of VR amnesia. Our explanation of how VR reduces memory is the same as our “attention distraction” hypothesis regarding how VR reduces pain perception. VR affects pain perception and memory for pain. Future research on the mechanism of how VR reduces memory for pain may enrich our understanding of how VR reduces subjective perceived pain intensity, VR analgesia.^2,35^

In conclusion, the current study demonstrates that immersive VR distraction significantly impairs memory for painful stimuli, supporting the hypothesis that immersive VR interferes with the brain’s ability to later remember physically painful experiences: VR-induced anterograde amnesia. This phenomenon could have profound clinical applications, particularly in potentially reducing the formation of unpleasant memories during painful medical procedures. Additional research is needed.

## DISCLOSURES

**Conflicts of Interest:** None. **Funding:** The current study was philanthropically funded by the Mayday Fund (VRAnalgesia2022) to the University of Washington, to H. G. Hoffman. The completion of this work was supported by Advanced Grant No. 101141285 of the European Research Council (#101141285) and Collaborative Research Center 1158 on Pain (grant no. B07) funded by the Deutsche Forschungsgemeinschaft to H. Flor. None of the funding agencies were involved in the study design, collection, analysis, interpretation of data, the writing of this article or the decision to submit it for publication. **This manuscript was handled by:** Anna Woodbury, MD.

## Supplementary Material
