# Supplementary material for: The Effect of Immersive Virtual Reality on Anterograde Amnesia and Subjective Pain During Procedures: A Within-Subject Randomized Controlled Study
Source: Anesth Analg. 2026 May 15;143(1):182–91. doi: 10.1213/ANE.0000000000007858 (PMC13249296; doi:10.1213/ANE.0000000000007858)
Supplement: Supplementary file 1 [file ane-143-182-s001.pdf]

Supplemental Digital Content 1.docx.

GRS ratings

How hard was it to concentrate on memorizing the pattern
of thermal pain stimuli during the most recent pain stimulus set (5 painful stimuli)

|____|
0 1 2 3 4 5 6 7 8 9 10

0	2	5	7	10
not difficult at all to concentrate	a little difficult/hard to concentrate	moderately difficult/hard to concentrate	very difficult/hard to concentrate	extremely difficult/hard to concentrate

How accurate is your memory for the painful stimuli during the most recent pain stimulus set
(5 painful stimuli in a row)

|____|
0 1 2 3 4 5 6 7 8 9 10

0	2	5	7	10
not accurate at all	a little accurate	moderately accurate	very accurate	extremely accurate

How distracted were you during the most recent pain stimulus set
(5 painful stimuli in a row), while trying to memorize the pattern of pain stimuli

|____|
0 1 2 3 4 5 6 7 8 9 10

0	2	5	7	10
not distracted at all	a little distracted	moderately distracted	very distracted	extremely distracted

Patient Global Impression of Change - Participants were asked to report their global impression of change on a 7- point scale from -3 to + 3 (-3 = very much worse, + 3 = very much better), Kamper et al., 2009

Compared to the accuracy of your memory for pain during No VR, how would you describe the accuracy of your memory for pain during Virtual Reality?

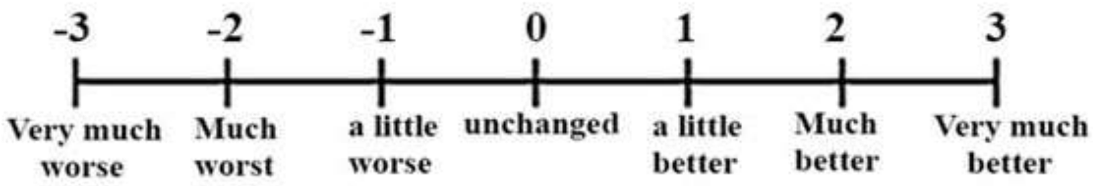

How much TIME did you spend thinking about your pain during the most recent pain stimulus set (5 painful stimuli)

I THOUGHT about my pain……

|____|
0 1 2 3 4 5 6 7 8 9 10

0	2	5	7	10
None of the time	some of the time	half of the time	most of the time	All of the time

How UNPLEASANT was your pain during the most recent pain stimulus set (5 painful stimuli)

|____|

0 1 2 3 4 5 6 7 8 9 10

not unpleasant at all	mildly unpleasant	moderately unpleasant	severely unpleasant	excruciatingly unpleasant

Rate your WORST PAIN during the most recent pain stimulus set (5 painful stimuli)

|____|

0 1 2 3 4 5 6 7 8 9 10

no pain at all	mild pain	moderate pain	severe pain	excruciating pain

How much FUN did you have during the most recent pain stimulus set (5 painful stimuli)

|____|

0 1 2 3 4 5 6 7 8 9 10

no fun at all	mildly fun	moderately fun	pretty fun	extremely fun

To what extent (if at all) did you feel NAUSEA (sick to your stomach) as a result of experiencing the virtual world duri
the most recent pain stimulus set (5 painful stimuli)

|____|

0 1 2 3 4 5 6 7 8 9 10

no nausea at all	mild nausea	moderate nausea	severe nausea	vomit

While experiencing the virtual world, to what extent did you feel like you WENT INSIDE the computer-generated worl
during the most recent pain stimulus set (5 painful stimuli)

|____|

0 1 2 3 4 5 6 7 8 9 10

I did not feel like I went inside at all	mild sense of going inside	moderate sense of going inside	strong sense of going inside	I went completely inside the computer-generated world

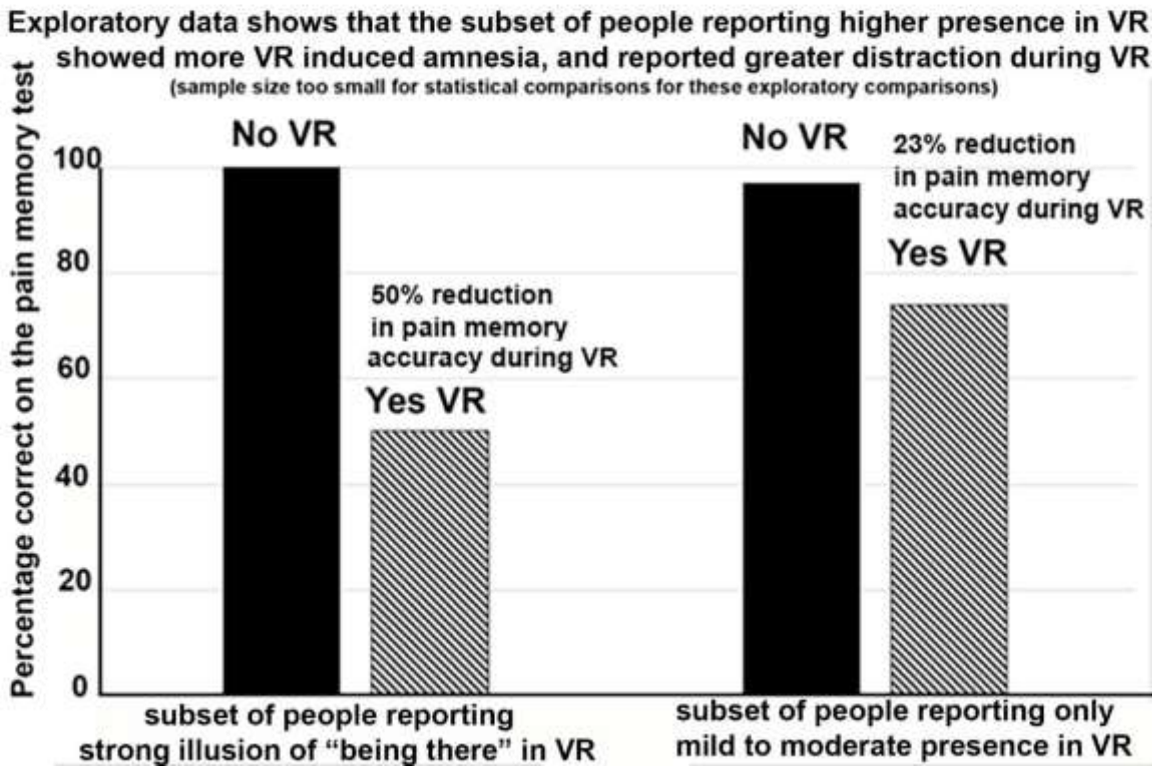

Figure A. Although the sample was too small for formal statistical analyses, descriptive results are as follows. Participants who reported a stronger illusion of "being there" in the computer-generated world as if it was a place they visited, showed larger VR induced amnesia: 50% reductions in pain memory accuracy, and they reported that it was very hard/difficult to concentrate on their pain during VR. In contrast, the subset of participants who reported only "mild to moderate" presence during VR showed only a 23% reduction in pain memory accuracy during VR, and they reported that it was only moderately difficult to concentrate during VR.
